# A summary of the current circumstances of migraineurs in China: a review of the GBD2019 database

**DOI:** 10.3389/fnhum.2025.1626607

**Published:** 2025-10-10

**Authors:** Changchang Ying, Qijun Yu, Qingling Zhai, Yonghui Pan

**Affiliations:** Department of Neurology, The First Affiliated Hospital of Harbin Medical University, Harbin, Heilongjiang, China

**Keywords:** migraine, incidence, disability, treatment, China

## Abstract

Migraine is a common, chronic and recurrent neurovascular disorder ranked by the WHO as one of the 20 most disabling conditions worldwide. Frequent and severe headaches can significantly impair patients’ quality of life, learning capacity, and physical and mental health. On the basis of the Global Burden of Disease (GBD) 2019 database, this article reviews the incidence, disability and treatment of migraine in China in recent years. This study aims to analyze the current status of migraine patients in China and to inform future research on its epidemiology, prevention, and treatment.

## 1 Background

Migraine is a neurovascular disorder primarily characterized by recurrent, unilateral, moderate-to-severe headaches. It is often accompanied by symptoms such as photophobia, phonophobia, nausea, vomiting and fatigue. According to the GBD 2019 report published by *The Lance* (GBD 2019 Diseases and Injuries Collaborators, 2020), headache disorders are the 14th leading cause of disability-adjusted life years (DALYs) globally among 369 diseases and injuries across 204 countries and territories. Notably, among women aged 15–49 years, it was the second leading cause of DALY. In 2019, headache resulted in disability for 46.6 million individuals worldwide, with migraine accounting for 88.2% of this burden, which is responsible for more disability than all other neurological disorders combined (Ashina et al., 2021). Therefore, enhancing the understanding of the epidemiology of migraine is fundamental for improving its diagnosis and treatment, as well as for the rational allocation of healthcare resources (Celentano et al., 1990).

This article reviews the incidence and contributing factors, disability and aggravating factors, and treatment of migraine in China in recent years. The analysis is based on data from the GBD 2019 and a literature search of databases, including PubMed and CNKI. Our methodology utilized data from the GBD 2019 study and a systematic literature search of databases, including PubMed and CNKI, for articles published between 2010 and 2024. The search keywords are listed in Supplementary Table 1. This work aims to characterize the current situation of migraineurs in China and to inform future research into its epidemiology, prevention, and treatment.

Current migraine research is often limited by small sample sizes, constrained study scopes, and high rates (Leonardi and Raggi, 2019). To address these limitations, our study prioritized the acquisition of robust and up-to-date epidemiological data. We therefore selected the GBD 2019 database as our primary data source. GBD 2019 combines literature studies, surveillance survey information, inpatient and outpatient data, health insurance status and other information to assess the incidence, prevalence, mortality and capacity-adjusted incidence of 369 diseases, injuries and 87 risk factors in 204 countries from 1990 to 2019 (GBD 2019 Diseases and Injuries Collaborators, 2020). This database can provide a wealth of information and up-to-date estimates on the burden of migraine at the global, regional and national levels (Murray and Lopez, 2013). The number of new migraine cases and age-standardized incidence rate (ASIR) were chosen as measures of migraine incidence from 2010 to 2019 on the basis of searches of the GBD database, with Num offering a visual representation of the number of new migraineurs during the year and ASIR offering a more comparable indication of migraine incidence. ASIR is expressed in terms of units per 100,000 people. DALY and age-standardized disability-adjusted life-year rates (ASDR) from the GBD database were selected as the disabling data. DALY is the number of years with any short- or long-term health loss. Both the DALY and the ASDR can measure disability rates and levels of disability.

The estimated annual percentage change (EAPC) is a summary of the trend. A regression line was fitted to the natural logarithm of the age-standardized rate (ASR) values; that is, y = α + βx + ϵ, where y = ln(ASR) and x = calendar year. The EAPC was calculated as 100 × [exp(β)–1], and its 95% confidence interval (CI) was obtained from a linear regression model (Liu et al., 2020). There is an increasing trend when both the EAPC estimate and the lower 95% CI are > 0 and a decreasing trend when both the EAPC estimate and the upper 95% CI are < 0. An EAPC with a 95% CI that included 0 was defined as a non-significant trend.

Locally weighted scatterplot smoothing (LOWESS) is a non-parametric regression method used to smooth scatterplot data. This method assigns a weight to each data point to minimize local regression residuals, thereby fitting the data locally without making global assumptions. It is particularly suitable for data exhibiting non-linear or complex patterns, effectively removing noise to reveal underlying trends (Cleveland and Devlin, 1988). The Shapiro–Wilk test revealed that the data for both ASIRs were non-normally distributed (*p* < 0.05), so LOWESS was chosen for analysis.

## 2 Incidence and causative factors

### 2.1 Incidence of migraine in China

As shown in Table 1, the number of new migraine cases in China decreased from 131,594,950 in 2010 to 129,397,650 in 2019. Conversely, the migraine ASIR in China increased from 938.02 per 100,000 people in 2010 to 961.69 per 100,000 people in 2019. The estimated annual percentage change (EAPC) for China’s ASIR was greater than 0 (lower limit of 95% CI > 0), confirming a statistically significant upward trend in incidence. This divergence indicates that although the absolute number of new cases declined, the underlying risk of developing migraine increased after adjustment for changes in population age structure and size. Unlike China, the ASIR showed a nearly stable pattern in Global, with an EAPC of 0.04% per year (95% CI: −0.00 to 0.09), where the lower bound of the CI slightly overlapped with zero, suggesting a non-significant change. The observed reduction in absolute case numbers may partly stem from China’s demographic transition, wherein a declining proportion of young adults—a group with higher susceptibility to new-onset migraine—may have counterbalanced the rising age-specific risk.

**TABLE 1:** Number of new migraine cases and age-standardized incidence rates (ASIRs) in China and Global, 2010—2019.

Location	Year	Number			Number change		ASIR			EAPC (ASIR)	
		Val	Lower CI (95%)	Upper CI (95%)	Val	95% CI	Val	Lower CI (95%)	Upper CI (95%)	Val	95% CI
China	2010	13,159,495	11,554,415	14,768,567	−0.02%	−0.06%∼0.02%	938.02	822.35	1050.93	0.16%	0.01%–0.31%
	2011	13,111,597	11,525,988	14,708,266			937.85	822.19	1050.75		
	2012	13,065,458	11,488,147	14,632,439			937.69	822.02	1050.59		
	2013	130,19,268	11,439,036	14,562,445			937.49	821.82	1050.41		
	2014	12,968,845	11,382,145	14,516,985			937.27	821.61	1050.22		
	2015	12,914,675	11,344,881	14,482,640			937.04	821.39	1050.02		
	2016	12,853,044	11282087	14441785			936.83	821.23	1049.84		
	2017	12,792,962	11,224,476	14,393,743			936.63	821.09	1049.66		
	2018	12,819,159	11,325,479	14,447,928			944.39	831.33	1059.72		
	2019	12,939,765	11,463,449	14,485,073			961.69	845.91	1079.18		
Global	2010	80,663,040	70,474,547	90,916,848	0.09%	0.07%∼0.1%	1133.80	986.91	1278.17	0.04%	−0.00%–0.09%
	2011	81,389,010	71,105,360	91,780,942			1134.06	988.58	1277.68		
	2012	82,100,095	71,744,694	92,566,596			1133.94	988.54	1278.44		
	2013	82,803,419	72,346,920	93,330,751			1133.65	986.37	1279.34		
	2014	83,498,589	72,903,751	94,051,051			1133.40	986.00	1279.23		
	2015	84,196,055	73,454,491	94,808,775			1133.36	985.89	1278.59		
	2016	84,875,529	74,048,285	95,546,230			1133.15	985.76	1278.18		
	2017	85,554,248	74,633,950	96,307,533			1132.85	985.50	1278.75		
	2018	86,472,817	75,639,117	97,355,419			1135.87	989.82	1282.12		
	2019	87,648,969	76,635,688	98,654,602			1142.54	995.90	1289.44		

**FIGURE 1:**
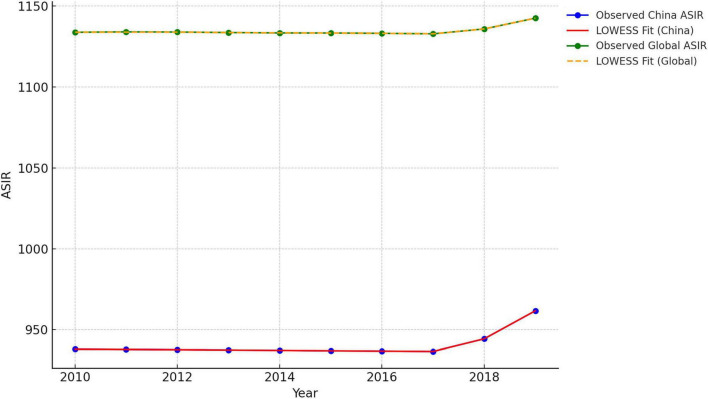
Comparison of age-standardized incidence rates (ASIR’S) locally weighted scatterplot smoothing (LOWESS) fitting: China VS Global, 2010–2019.

To further compare the ASIRs, we used LOWESS to analyze the ASIRs for China and globally from 2010 to 2019, and the results are shown in Figure 1. The key parameter in LOWESS is the smoothing fraction (frac), which controls the amount of smoothing. In this case, we set frac = 0.3, meaning that each data point is smoothed using 30% of the nearest points. This parameter was chosen to ensure adequate smoothing while capturing local fluctuations in the data. The residual plots for both the Chinese and global datasets demonstrate that the residuals are centered around zero with no systematic patterns, suggesting that the LOWESS model appropriately captures the underlying trends (shown in Supplementary Table 2).

Figure 1 presents the LOWESS smoothed curves for both China and the global ASIR, with visual indications of a potential turning point approximately 2017. Both datasets show relatively stable trends before 2017, followed by noticeable upward shifts after 2017. To assess the change in trend, we estimated the slope of the LOWESS smoothed curves before and after 2017 (Table 2). The slope was computed via simple linear regression for both China and the global ASIR. The China ASIR exhibits a sharp and more pronounced shift, with the slope increasing by approximately 12.73 units per year. In contrast, the global ASIR shows a more moderate change, with an increase of 4.98 units per year. To test whether the observed change in slopes was statistically significant, we performed a paired t-test to compare the slopes before and after 2017 for both China and the global ASIR (Table 2). Both regions have *p*-values < 0.01, indicating that the change in slope after 2017 was statistically significant. This strongly supports the conclusion that the observed shift in ASIR trends is not due to random fluctuations in the data. The reason for the turning point in 2017 may be related to the new migraine diagnostic criteria published in 2018 (ICHD-3) (Cephalalgia, 2018). The new migraine diagnostic criteria are more comprehensive than the previous diagnostic criteria are, which may have improved case identification and contributed to the recorded increase in incidence. However, why the incidence of migraine continued to rise after 2017 is a question worth pondering.

**TABLE 2:** Slopes of the locally weighted scatterplot smoothing (LOWESS) curves for China and Global age-standardized incidence rate (ASIR) before and after 2017.

Location	Slopes of the LOWESS curves		t-statistic	*P*-value
	Period	Val		
China	2010–2017	−0.2004	9.96	< 0.01
	2017–2019	12.5300		
Global	2010–2017	−0.1389	5.92	< 0.01
	2017–2019	4.8450		

In addition, the ASIR in China is much lower than it is globally, as shown in Figure 1. To determine whether China’s data are normal, we choose five categories of ASIRs classified by the SDI. The SDI is a composite indicator of the development status of a country/region.

**FIGURE 2:**
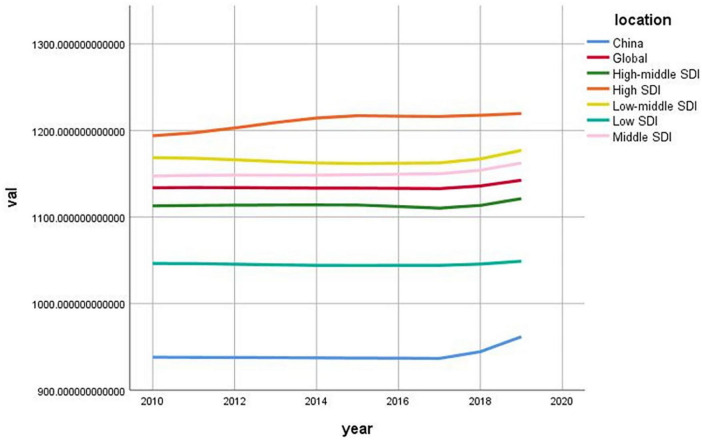
Age-standardized incidence rate (ASIR) of migraine in China, Global and five sociodemographic index (SDI) categories/regions, 2010–2019.

There are five categories: high-SDI countries/regions (mainly developed countries such as the United States and France), high-middle-SDI countries/regions (mainly countries such as Argentina, Belarus and Chile), middle-SDI countries/regions (mainly developing countries such as China and Brazil), medium-low-SDI countries/regions (mainly countries such as India and Egypt), and low-SDI countries/regions (mainly countries such as Afghanistan and Somalia). Through the SDI, we can obtain an initial general picture of ASIRs worldwide for countries at several levels. When the trends in the ASIRs for these five categories of SDI countries/regions, China and the globe are compared, it seems that there is no exact positive association between the SDI and the ASIR for migraine (Figure 2). Although China is a medium-SDI country, the ASIR in China seems significantly lower than that in medium-SDI countries and even lower than that in low-SDI countries (*P* < 0.001, Table 3), which is highly anomalous. Considering the composition of the GBD2019 data, this could mean that a significant number of Chinese migraineurs are not diagnosed, treated, or enrolled, so its actual reference value is debatable. A review of the trend of ASIRs in China from 2010 to 2019 revealed that ASIRs are significantly greater in China than they are globally, which may be related not only to the significant increase in the incidence of migraine in China but also to the development of China’s economy, the popularization of medical knowledge, and the increase in people’s awareness of the disease, which has led to the emergence of implicit data on the incidence of migraine.

**TABLE 3:** Age-standardized incidence of migraine in China and low-sociodemographic index (SDI) categories/regions, 2010–2019.

Group	M (P25,75)*	Difference in medians (95% CI)^#^	Wilcoxon two-sample rank-sum test	
			Z value	*P*-value
China	937.590 (936.911,939.614)	107.308 (106.190–108.451)	3.780	< 0.001
Low SDI	1045.114 (1044.156,1046.173)			

*Since neither China’s nor the low-SDI countries’ 2010–2019 incidence rates were normally distributed data, the median of the ten years was taken for comparison, and data located on the 1/4 and 3/4 were also chosen as the intervals. ^#^Difference between the median incidence rate in low-SDI countries and in China, with 95% confidence intervals.

#### 2.1.1 Effects of sex on the incidence of migraine in China

**FIGURE 3:**
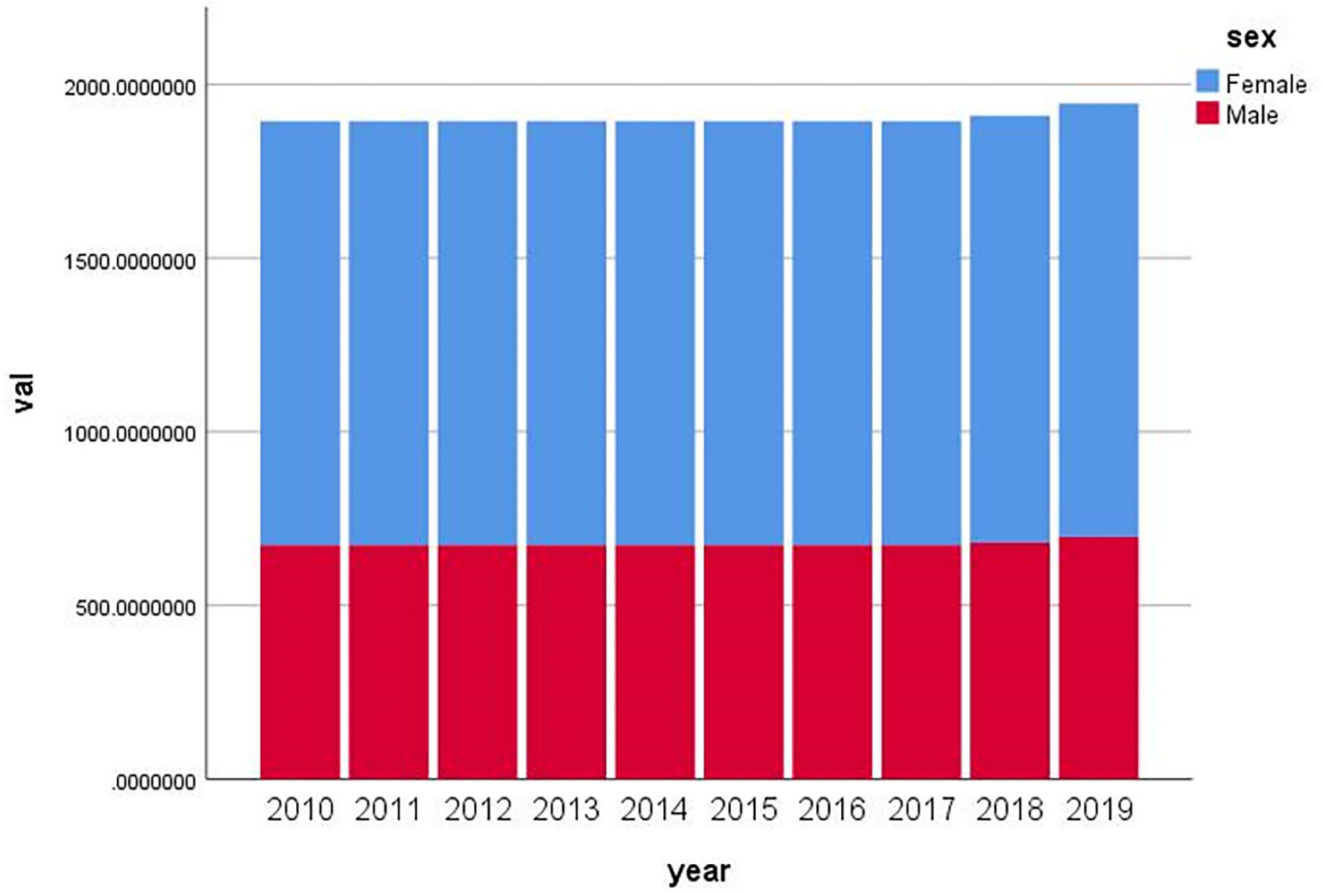
Histogram of age-standardized male and female migraine incidence rates, China, 2010–2019.

On the basis of data from the GBD2019 database, we plotted a histogram of age-standardized male and female migraine incidence rates in China from 2010 to 2019 (Figure 3). The incidence of migraine in China is increasing in both males and females but is consistently much higher in females than in males. A study performed by Wang et al. (2022), Liu and Tan (2014) analyzed the incidence of sex- and age-differentiated migraine in China from 1990 to 2019 using point and age-period cohorts and concluded that the incidence of migraine in China is consistently much higher in females than in males.

The reasons why women are more likely to experience migraine are complex. Smitherman and Ward (2011) reported that women are more likely to report pain than men are because they are more sensitive to noxious stimuli and have higher pain ratings and lower pain thresholds than men are. In addition, Celentano et al. (1990) noted that migraine attacks are more severe in women than in men, leading to more severe impairment and longer recovery periods. In terms of physiology, Stewart et al. (1992) noted that most female patients develop migraine at the onset of menstruation, with the incidence peaking at ages 30 and 40 years and decreasing significantly after menopause. David et al. (2014) reported that sex hormone changes during puberty, pregnancy, lactation and menopause are the main triggers for the development of migraine in women. The estrogen withdrawal hypothesis proposes that menstrual migraine is caused by a sudden drop in estrogen during the late luteal phase prior to menstruation and that the onset and development of migraine in women are influenced by ovarian hormones (MacGregor and Hackshaw, 2004). Lifestyle has an impact on migraine, but it affects men and women differently. For example, dietary sodium intake is strongly associated with the incidence of migraine in women but not in men (Pogoda et al., 2016). Women are more likely to experience higher levels of psychological burden than men. Epidemiological studies indicate that women have a higher lifetime risk of developing psychiatric disorders (such as depression and anxiety) than men do (Smitherman and Ward, 2011).

**FIGURE 4:**
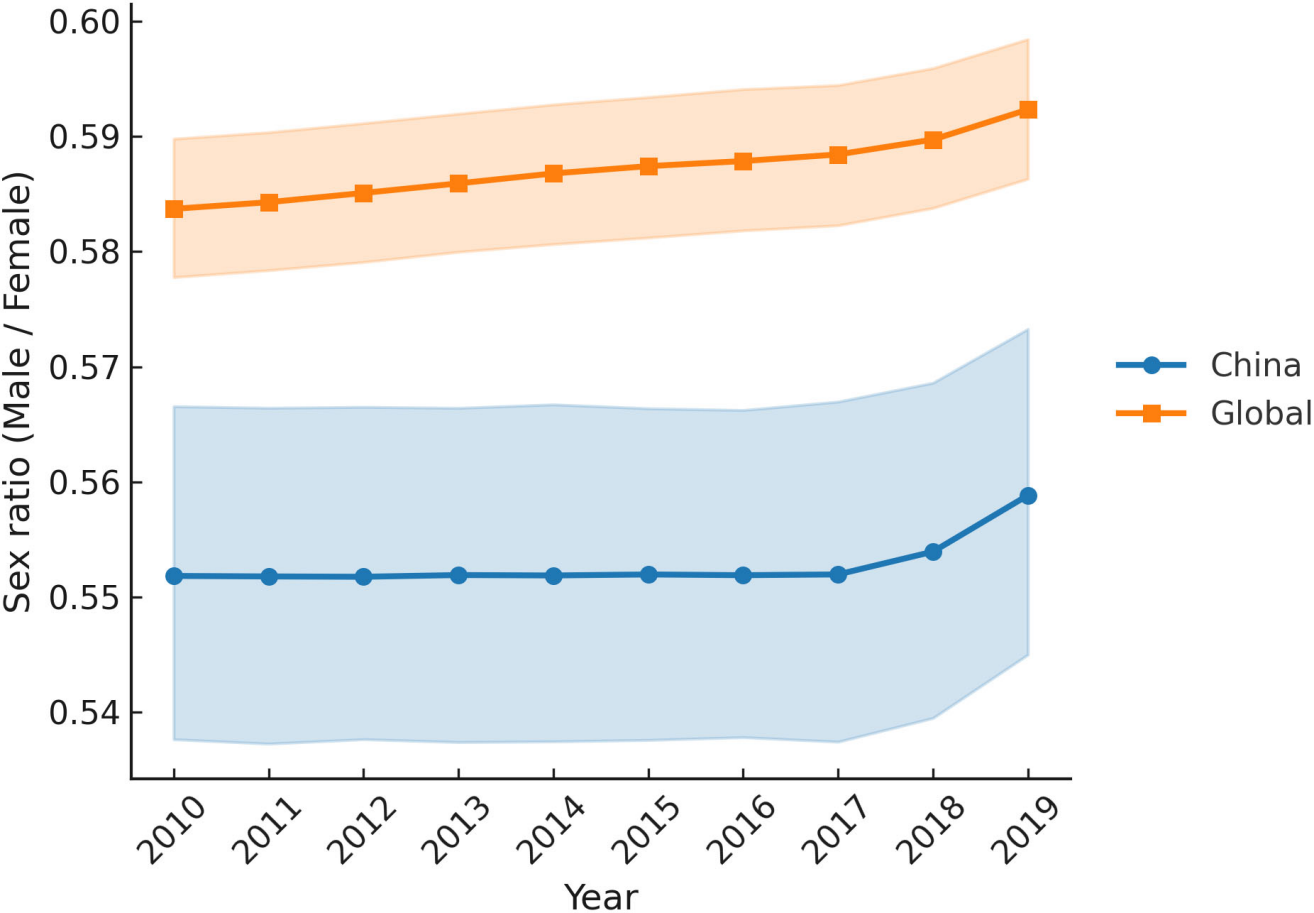
Migraine incidence sex retio (male/famale): China VS Global, 2010–2019.

To further elucidate the sex differences in migraine incidence in China, we produced age-standardized male and female incidence rates and incidence sex ratios (male/female) for migraine in China and Global from 2010 to 2019 on the basis of data from the GBD2019 (Figure 4). The bootstrap analysis of age-standardized incidence rates demonstrated a consistent female predominance in migraine for both China and the global population between 2010 and 2019. The sex ratio (male/female) remained stable across years, with median values of approximately 0.55 (95% CI ≈ 0.54–0.56) in China and 0.58 (95% CI ≈ 0.57–0.59) globally. Year-to-year variability was minimal, with narrow confidence intervals indicating stable estimates over time. When the data from 2010 to 2019 were aggregated, the overall male-to-female ratio was 0.55 (95% CI: 0.54–0.56) for China and 0.58 (95% CI: 0.57–0.59) for the global population. The non-overlapping confidence intervals suggest a statistically significant difference, indicating that Chinese women experience a disproportionately greater burden of migraine than men do—a disparity that is more pronounced in China than Global. Genetic factors may contribute to these patterns. For example, Xingkai (2021) reported that variants in the ESR1 gene are associated with the development of migraine in Chinese patients. ESR1 rs2234693 is a risk factor for migraine without aura, female migraine, and menstrual-associated migraine. Nevertheless, the underlying causes of the increased susceptibility to migraine among Chinese women warrant further investigation.

Overall, these findings highlight the need for sex-specific approaches for migraine prevention and management. In particular, targeted strategies for women—especially in high-burden settings such as China—could contribute to reducing the population-level impact of migraine.

#### 2.1.2 Effect of age on the incidence of migraine in China

**FIGURE 5:**
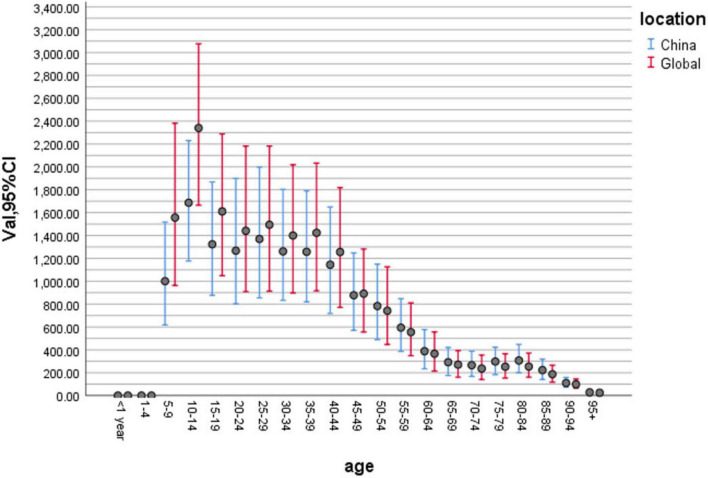
Histogram of the age distribution of migraine incidence in China and Global in 2019.

To compare the differences in migraine incidence across different age groups, we constructed a bar chart of the age distribution of migraine incidence in China and globally in 2019 on the basis of relevant data from the GBD2019 (Figure 5). We can see that 10–14 years of age is the period with the highest incidence of migraine in China, followed by 25–29 years of age. The incidence of migraine in China remains high in the age range of 10–44 years. Wang et al. (2022) reported that the incidence of migraine in China was highest in those aged 10–14 years, followed by those aged 22–44 years in both sexes. An epidemiological study in Shanghai (Jin et al., 2013) revealed that the incidence of migraine in children and adolescents varied with age between 13% and 43%, with the highest incidence of migraine occurring in 14–15 year-olds. The age range of 10–19 years is adolescence, and the enormous psychological transition and greater academic pressure may explain the high incidence of migraine at this age. Additionally, when we compared the age distributions of migraine incidence in China and globally in 2019, we were surprised to find that incidence rates in China were always lower than those globally at the age of 5–49 but always higher than those globally at the age of 50 years and older.

### 2.2 Disability and factors

#### 2.2.1 Disability in migraine patients in China

As shown in Table 4, the DALY due to migraine in China increased from 6,732,178 (1,056,709, 15,187,073) in 2010 to 7,089,417 (1,130,496, 16,097,220) in 2019, an increase of 0.05% (0.01%–0.10%). The number of migraine-related DALYs worldwide increased from 37,529,229 (6,104,192, 85,300,123) in 2010 to 42,077,666 (6,295,417, 95,645,211) in 2019, an increase of 0.12% (0.11%∼0.14%).

**TABLE 4:** Disability-adjusted life year (DALY) and age-standardized disability-adjusted life years rate (ASDR) for migraine in China and Global, 2010–2019.

Location	Year	DALY			DALY change		ASDR			EAPC (ASDR)	
		Val	Lower CI (95%)	Upper CI (95%)	Val	95% CI	Val	Lower CI (95%)	Upper CI (95%)	Val	95% CI
China	2010	6,732,178	1,056,709	15,187,073	0.05%	0.01%∼0.10%	425.22	64.66	958.26	0.15%	−0.02%–0.32%
	2011	6,770,960	1,060,981	15,215,772			425.25	63.90	958.05		
	2012	6,805,724	1,071,389	15,346,848			425.24	64.47	956.55		
	2013	6,836,876	1,081,889	15,460,419			425.17	64.30	957.00		
	2014	6,863,400	1,095,946	15,461,917			425.11	64.22	957.79		
	2015	6,886,143	1,096,625	15,505,814			424.50	64.17	956.40		
	2016	6,902,580	1,107,125	15,521,467			424.83	64.12	955.13		
	2017	6,916,781	1,114,494	15,575,593			424.75	64.48	955.62		
	2018	6,976,016	1,125,059	15,765,518			427.99	64.08	964.43		
	2019	7,089,417	1,130,496	16,097,220			435.42	63.68	991.80		
Global	2010	37,529,229	6,104,192	85,300,123	0.12%	0.11%∼0.14%	522.06	80.04	1187.16	0.03%	−0.02%–0.09%
	2011	38,027,288	1,096,625	86,456,498			522.15	79.85	1187.20		
	2012	38,517,540	1,114,494	87,763,495			522.18	79.93	1189.89		
	2013	38,999,958	6,199,903	88,820,242			522.15	79.87	1189.99		
	2014	39,472,769	6,418,383	89,938,506			522.08	79.66	1190.15		
	2015	39,941,303	6,359,230	90,957,933			522.00	79.40	1191.41		
	2016	40385053	1130496	91893869			521.65	79.61	1187.76		
	2017	40,824,502	1,107,125	93,050,087			521.31	79.39	1187.83		
	2018	41,385,223	1,125,059	94,028,778			522.56	79.36	1189.20		
	2019	42,077,666	6,295,417	95,645,211			525.54	78.79	1193.99		

The estimated annual percent change (EAPC) in the ASDR of migraine patients from 2010 to 2019 is also presented in Table 4. In China, the EAPC was + 0.15% per year (95% CI: −0.02% to +0.32%, *p* = 0.073), indicating a slight but statistically non-significant increase. At the global level, the EAPC was + 0.03% per year (95% CI: −0.02% to + 0.09%, *p* = 0.197), also suggesting no significant change. Overall, both China and the world exhibited stable ASDR trends during the study period, with no evidence of substantial increases or decreases.

This observed stability may be attributed to several factors. First, as a chronic neurological condition, migraine is characterized by high prevalence and extended disease duration; however, it generally has limited short-term variation in population-level burden. Consequently, the DALY rate remains relatively consistent over time. Second, although diagnostic capabilities and treatment access have improved over the past decade, these advances have not yet translated into substantial reductions in the overall burden of migraine at the population level. Notably, effective preventive therapies—such as CGRP monoclonal antibodies—became available only toward the end of the study period (after 2018) and thus had minimal influence on the trends observed herein. Finally, the slightly higher—although not statistically significant—EAPC observed in China compared with the global average may be associated with ongoing urbanization and lifestyle-related risk factors; however, the effect remained too small to reach statistical significance.

#### 2.2.2 Factors influencing the degree of disability in migraine patients in China

Migraine can lead to widespread health loss, reduced quality of life and decreased productivity. GBD 2019 reported that migraine is the 2nd leading cause of disability-related loss of healthy life years in Greece and the 5th leading cause in China (GBD 2019 Diseases and Injuries Collaborators, 2020). Therefore, analyzing the factors affecting the degree of disability associated with migraine could facilitate further interventions to reduce its disability burden.

The migraine disability assessment questionnaire (MIDAS) (National Institute for Health and Care Excellence, 2021) is currently the most commonly used clinical tool for assessing migraine disability and focuses on three domains of lost time: study or paid work, housework, and family, social or leisure activities. Additionally, in recent years, the Headache Impact Test-6 (HIT-6) (Stewart et al., 2001) has been widely used in China to complement the assessment of disability in migraine patients. This scale measures the adverse effects of migraine on social functioning, vitality, role functioning, psychological distress, attention, and headache severity.

The severity of migraine, the comorbidity of migraine with psychiatric disorders, the type of migraine, the presence of prodromal symptoms in migraine, and the continuity of clinical follow-up treatment all influence the degree of disability in migraine patients.

##### 2.2.2.1 Severity of migraine

The severity of migraine refers to the intensity of headache, the frequency of headache attacks and the duration of each attack. In a retrospective study of 94 patients in Hubei, Zhang and Huang (2016) reported that the severity, frequency and duration of attacks were the main causes of functional disability in patients. In a multifactorial analysis of 94 migraine patients in Xi’an, Luo et al. (2012) reported that the more frequent the headache attacks were, the more severe the headache and the longer the duration of each attack, the more severe the migraine-related functional disability. The results also revealed that MIDA scores were positively associated with headache attack frequency and duration, whereas HIT-6 scores were positively associated with headache severity, anxiety/depression, and attack frequency (Luo et al., 2012). The correlations between the number of days of headache and MIDAS scores and between headache severity and HIT-6 scores were greater (Yuan and Chuang, 2021).

##### 2.2.2.2 Comorbidity of migraine and psychiatric disorders

Migraine and psychiatric disorders have a high degree of comorbidity. Migraine and depression constitute up to 20% of the common causative genes (Yin and Xu, 2010). Fan et al. (2019) concluded that although the effects of migraine and depression are bidirectional, migraine is more likely to cause depression.

When migraineurs suffer from anxiety, depression and sleep disorders, the probability of disability due to migraine increases, which has a serious impact on the quality of life of patients. Long-term, recurrent, severe migraine attacks and psychiatric symptoms such as anxiety, depression and sleep disorders are mutually reinforcing, resulting in a vicious cycle (Luo et al., 2012), which may eventually lead to suicide. The ability of migraine to cause depression can be indirectly disabling. A study by Zhou and Yu (2015) noted that depressive disorders are also a significant cause of disability worldwide, and by 2020, major depression is expected to be the second largest burden of disease after ischemic heart disease. Severe headache, a long duration of illness, poor sleep quality, a positive family history of headache and low life satisfaction are the main risk factors for comorbid anxiety/depression in migraine patients (Luo et al., 2012).

Migraine attacks are often accompanied by a variety of psychosomatic symptoms, such as anxiety and irritability, depression, agitation, panic, bipolar affective disorder, and a variety of sleep disorders, such as difficulty falling asleep, excessive dreaming, and difficulty maintaining sleep. Fan et al. (2019) selected 78 migraine patients for the MIDAS score and HIT-6 score. The results of the present study revealed that the prevalence rates of anxiety and depression in migraine patients were 48.72% and 51.28%, respectively, which were significantly greater than those reported in the healthy population. Xia and Zhang (2014) reported that migraine patients generally had poor sleep quality, which was positively correlated with anxiety and depression in 167 migraine patients according to the Pittsburgh Sleep Quality Inventory and the Hospital Anxiety and Depression Scale, respectively. Luo et al. (2012) noted that when studying the psychosomatic symptoms associated with migraine, the assessment of anxiety, depression and sleep quality could be considered as a whole to reflect the comorbidity of migraine-related psychiatric disorders in a more comprehensive manner.

##### 2.2.2.3 Types of migraine

Migraine can be classified into episodic migraine (EM) and chronic migraine (CM) on the basis of the frequency of attacks. EM is defined as having fewer than 14 migraine attacks per month; CM is defined as having ≥ 15 days of headache per month, with at least 8 days of attacks conforming to migraine characteristics, for at least 3 months. Each year, 2.5% of EM patients convert to CM (Blumenfeld et al., 2011). Many risk factors contribute to the chronicity of migraine, with low socioeconomic status and low educational attainment being the two most significant risk factors (Carod-Artal et al., 2012). Interestingly, depression and anxiety disorders can also contribute to migraine chronicity (Seng et al., 2017). Compared with EM patients, CM patients are more likely to have more severe disability, lower health-related quality of survival, higher levels of anxiety and depression and higher healthcare resource utilization (Fan et al., 2019). A study by Yang et al. (2018) also revealed that CM patients were more severely affected in terms of headache levels, social functioning, vitality, role functioning, psychological distress, and attention. Of these, social functioning, vitality, and attention were more prominently affected. Furthermore, in contrast to the findings of a study in a developed country (Blumenfeld et al., 2011), CM patients were more likely to miss time for work, study and housework, whereas Chinese CM patients persisted in attending work, study and housework even when they were inefficient (Yang et al., 2018). This may be related to the national situation in China, as a developing Chinese country with a large population, the strength of social security cannot be compared to that of developed countries, and most CM patients cannot give up their social activities to get paid, even if they are inefficient due to headaches.

##### 2.2.2.4 Prodrome symptoms

The migraine stages include the prodrome, aura, headache, and recovery phases. The prodromal phase is defined as a state in which a group of non-painful symptoms occur frequently hours to days before the onset of headache symptoms. These symptoms include sleep-related symptoms, appetite and eating-related symptoms, mood changes, inattention, neck stiffness, gastrointestinal symptoms, increased sensitivity to external stimuli, and vestibular symptoms (Xu et al., 2021). In a retrospective analysis of 107 patients in Shanghai, Xu et al. (2021) noted that migraine patients with prodromal symptoms had higher levels of pain and more impaired quality of life than those without prodromal symptoms. The study also revealed that 85.29% of patients with prodromal symptoms had a high warning rate (> 60%) for headache, and 57.36% had a 90% warning rate, suggesting that prodromal symptoms may have high warning value for migraine.

##### 2.2.2.5 Continuous clinical follow-up treatment

In a study by Liu and Tan (2014), a longitudinal comparison of the World Health Organization Disability Assessment Scale II (WHO-DAS II) and Migraine-Specific Quality of Life Questionnaire 2.1 (MSQ2.1) scores after 3 months of follow-up treatment in 118 newly seen patients in China revealed that patients who adhered to follow-up treatment improved (fewer headache frequency and a reduction in acute medication use), patients’ quality of life improved, and disability decreased. The MSQ2.1 scores of the patients significantly changed (ElasticSearch val = 0.57) in the “functional limitation” dimension. Positive changes were observed in the “loss of function” and “emotional functioning” dimensions and in the total score, but they were less significant than they were in the “functional limitation” dimension (Liu and Tan, 2014). This finding contrasts with those of similar studies abroad and may be related to differences in the economic and cultural lives of people at home and abroad, among other factors.

### 2.3 Treatment of migraine in China

#### 2.3.1 Status of treatment in China

A study by Zhao et al. (2023) assessed the treatment patterns of Chinese EM patients via real-world data from the Adelphi Migraine Disease-Specific Project (DSP). Data were drawn from an existing dataset, the Adelphi Migraine DSP, which is a point-in-time survey conducted in China (January–June 2014) (Zhao et al., 2023). Notably, 86.1% of the Chinese patients were on ante meridiem (AM) drugs (NSAIDs: 62.7%, tretinoin: 7.7%), 38.5% were on post meridiem (PM), and 24.9% were on both. Approximately 55% of patients had ≥ 1 problem with current AM or PM, and migraine-related symptoms (including nausea, photophobia, and phonophobia) were completely controlled in < 5% of patients receiving NSAIDs (50.21%–7.38%) or tretinoin (4.32%–4.43%). A total of 2.3% of patients reported an inadequate response to the current AM (migraine in ≤ 5/42 episodes resolving completely within 5 h). A total of 2.3% of patients reported an inadequate response to current AM (migraine in ≤ 5/42 episodes resolving completely within 5 h) (Zhao et al., 2023). A study by Takeshima et al. (2019) revealed that among migraineurs from China, 52.9% to 68.6% had previously consulted a physician, 37.2%–52.7% of those diagnosed with headache had not previously been diagnosed with migraine, and 13.5%–18% had previously been diagnosed with migraine.

The awareness and diagnosis of migraine in China remain suboptimal, characterized by a low symptom consultation rate (approximately 52.9%) and an even lower correct diagnosis rate (approximately 13.8%) (Liu et al., 2013). Although historical population-based surveys have established a foundational understanding of migraine prevalence and healthcare utilization patterns, a significant portion of this data is now over 10 years old (Liu et al., 2013; Yu et al., 2012). More recent real-world evidence, derived from claims and prescription databases, indicates that only approximately one-quarter of patients with migraine receive acute prescription medications (Huang et al., 2024; Yu et al., 2020). Furthermore, the prescription of triptans—a mainstay of specific migraine therapy—remains exceptionally low at around 3.3% (Yu et al., 2020). Collectively, these findings underscore two critical issues: the constrained representativeness of existing epidemiological data and the pressing need for contemporary, large-scale studies to inform effective public health strategies and clinical management for migraine in China.

#### 2.3.2 New advances in China

There is no definite conclusion about the pathogenesis of migraine, which may be related to genetic, neurological and vascular factors. The neurological theory, the vascular origin theory and the trigeminal vascular theory are the three main current theories, among which the trigeminal vascular theory is now generally considered the most likely mechanism (Zhang et al., 2017). The trigeminal vascular theory suggests that migraine attacks are closely related to the trigeminal vascular reflex and that the pathogenesis involves neuropeptide release, intracranial vasodilatation and altered permeability due to activation of the trigeminovascular system (TGVS) and the development of aseptic inflammation (Thuraiaiyah et al., 2022). New advances in migraine treatment in China are divided into three main areas: medication and neuromodulation therapy and Chinese medicine.

##### 2.3.2.1 Pharmacological treatment

Pharmacological treatment of migraine mainly consists of acute attack treatment and prophylaxis. NSAIDs, antihistamines, opioid sedatives and tricyclic antidepressants are commonly used to treat migraine but are non-specific and associated with certain gastrointestinal adverse effects (Guo, 2022). Tretinoin, ergotamine and fluoxetine are conventional specific agents and are mostly used as first-line treatments for acute attacks of moderate to severe migraine, but they are contraindicated in patients with coronary artery disease and brainstem aura, which is a major limitation (Guo, 2022). Moreover, β-blockers, calcium channel blockers and antiepileptic drugs are traditional prophylactic drugs, and their frequent use may increase the risk of overdose headache, which in turn affects the quality of life of patients (Guo, 2022). Therefore, the development of safer and more effective specific drugs for treating migraine has become an urgent issue.

The development of calcitonin gene-related peptide (CGRP)-related drugs has been a major development in the treatment of migraine in recent years. The use of CGRP receptor antagonists [gepants, such as ubrogepant, rimegepant, and zavegepant (Pellesi et al., 2025)] and CGRP monoclonal antibodies provides more options for the clinical management of migraine. The former can be used as an acute target. The evidence indicates that these agents provide efficacy comparable to that of NSAIDs but with superior tolerability and minimal cardiovascular risk (Pellesi et al., 2024; Pellesi et al., 2025). The latter can be used as a prophylactic treatment, which is not only efficacious but also has fewer adverse effects and low cardiovascular risk, greatly reducing the overuse of drugs and slowing the chronic progression of migraine (Takeshima et al., 2019). Despite these advances, key limitations persist. The long-term safety of CGRP-based therapies remains incompletely characterized, particularly in vulnerable populations such as children, pregnant women, and older adults (Pellesi et al., 2024).

In the Chinese chronic migraine population, atogepants are being studied for efficacy, safety and tolerability (Zhang et al., 2017). In contrast, atogepant is the first oral CGRP receptor antagonist drug used for the prophylactic treatment of migraine and has high popularity value. The results of current overseas clinical trials show that the drug is effective in reducing the average number of migraine days per month in subjects, with nausea and constipation being the most common adverse drug reactions and no significant hepatic adverse reactions or drug interactions (Yang, 2022).

To date, only a limited number of CGRP-targeted agents have been officially approved in China. Among these, erenumab was the first monoclonal antibody against the CGRP receptor to receive approval in September 2023 for the preventive treatment of migraine. It was followed by galcanezumab in January 2024. More recently, in January 2024, rimegepant—an oral CGRP receptor antagonist (gepant)—was approved for both acute and preventive treatment, making it the first dual-indication CGRP antagonist available in China. Compared with that in other high- and middle-income countries, the approval of CGRP-targeted agents in China is relatively recent. For context, erenumab and galcanezumab were first approved in the United States in 2018 and subsequently in the European Union later that same year. Rimegepant received initial FDA approval in February 2020 for acute migraine treatment, with an additional preventive indication granted in 2021, and was approved in the European Union in April 2022.

Despite these advancements, the availability of CGRP-related therapies in China remains limited compared with that in many other countries. Furthermore, several other novel migraine treatments—such as lasmiditan—have been approved abroad. Further clinical trials are needed to evaluate their suitability and efficacy in the Chinese patient population.

##### 2.3.2.2 Neuromodulation therapy

Neural network dysregulation is an important part of the pathogenesis of migraine, and early studies have shown that stimulating innervated areas around the trigeminal nerve can relieve migraine symptoms (Hou et al., 2022). In recent years, a variety of neuromodulation treatments targeting different peripheral and central nerve sites have emerged and have shown good efficacy, and neurostimulation has become a promising modality for the treatment of headache disorders (Ferrari et al., 2022).

A randomized double-blind placebo-controlled trial of Cefaly, a portable transcutaneous low-frequency electrical nerve stimulator, demonstrated that transcutaneous electrical stimulation of the supraorbital branch of the trigeminal nerve can relieve migraine symptoms and is effective and safe for mild, moderate and severe attacks (Kuruvilla et al., 2022). Cefaly was approved by the Chinese Food and Drug Administration as a Class II medical device for the prevention and treatment of migraine.

As migraine has a similar neuropathological basis to that of epilepsy and psychiatric disorders, some physiotherapy treatments that are effective for epilepsy or mood regulation are also effective for migraine patients. Transcranial magnetic stimulation (TMS) therapy is based on the principle of electromagnetic induction, where a magnetic field penetrates the skull to produce a series of physiological and biochemical responses that modulate neural activity, and has been widely used to treat epilepsy and psychiatric disorders (Somaa et al., 2022). In 2017, TMS of the occipital region of the brain was approved by the Food and Drug Administration (FDA) for migraine treatment. therapy was approved by the FDA for the acute treatment and prevention of migraine (U.S. Food and Drug Administration, 2013).

Another neuromodulation therapy, fastigial nucleus stimulation (FNS), inhibits inflammatory responses in ischemic areas of the brain, inhibits excitotoxic damage to neurons, inhibits neuronal apoptosis in ischemic areas of the brain, promotes regeneration and reconstruction of neural tissue, and increases cerebral blood flow, thereby treating ischemic stroke; FNS can also reduce the level of inflammatory cytokines in brain tissue and improve poststroke depression (Yu and Zhang, 2022). The mechanism of action of FNS suggests that FNS should also have some efficacy in migraine, but there is a lack of certain clinical trials to confirm this.

In recent years, new neuromodulation devices and methods have been introduced into clinical practice, and these devices are becoming increasingly widely used to treat migraine because of their reliable efficacy and progressive development toward miniaturization and portability. Although many neuromodulation devices have been shown to be effective in the treatment of migraine, the response to different devices varies from patient to patient, and high-quality research evidence is needed to form a definitive recommendation (Yuan and Chuang, 2021).

##### 2.3.2.3 Traditional Chinese medicine

The unique advantage of traditional Chinese medicine in the prevention and treatment of migraine is that it can be used in accordance with the concept of “treating the symptoms when it is urgent and treating the root cause when it is slow,” and it can also be used in a flexible combination of various means, including traditional Chinese medicine, acupuncture, moxibustion, moxibustion, pressure points, massage, an acupuncture knife and fire cupping, to achieve individualized treatment (An et al., 2022). The latest Chinese clinical practice guidelines for migraine (2020) (Xu et al., 2020a) state that migraine patients should be treated in stages, with acupuncture being the mainstay of acute treatment and medication being the mainstay of preventive treatment.

Acupuncture is currently a research hotspot for non-pharmacological treatment of migraine in China. Studies have shown that acupuncture induces cortical responses in migraine patients and that both the medial and peripheral conduction pathways of nociception and the nociceptive downstream regulatory system are involved in the modulation of nociception in migraine patients (Ma et al., 2021). A 24 weeks randomized controlled trial included 249 migraine patients without aura who were randomized into a true acupuncture group (83 patients), a sham acupuncture group (83 patients) and a control group (usual care, 83 patients) and were treated for 4 weeks with a 20 weeks follow-up. The reduction in the number of headache attacks was greater in the real acupuncture group (95% CI: 0.400–1.900, *P* = 0.002) and the control group (95% CI: 1.100–2.500, *P* < 0.001) than in the sham acupuncture group (95% CI: 1.100–2.500, *P* < 0.001), indicating that acupuncture can effectively reduce the number of migraine attacks (Zhao et al., 2017). Another multicenter randomized controlled clinical trial included 150 migraine patients without aura who had not been treated with acupuncture and were randomized to a manipulative acupuncture group (60 patients), a sham acupuncture group (60 patients) and a control group (usual care, 30 patients) after a 4 weeks baseline assessment, 8 weeks of treatment and 12 weeks of follow-up. The results revealed that the duration of headache attacks was significantly reduced by 1.4 days (95% CI: 2.400–0.300, *P* = 0.005) at weeks 13–16 and 2.1 days (95% CI: 2.900–1.200, *P* < 0.001) at weeks 17–20 after randomization compared with the sham acupuncture group, indicating that manipulative acupuncture treatment significantly reduced the duration and frequency of migraine attacks (Xu et al., 2020b). Owing to its efficacy and safety, traditional Chinese medicine for migraine has gradually gained the attention and recognition of the Western medical community in recent years, as the “Westernization of secondary school” has gained momentum. Migraine sufferers could be treated with 10 courses of acupuncture over a 5–8 weeks period.

Although Chinese medicine is not yet widely recognized internationally, several studies have shown that Chinese medicine is also effective in the treatment of migraine. The latest Chinese clinical practice guidelines for migraine in Chinese medicine (2020) (Xu et al., 2020a), after summarizing a large number of randomized controlled clinical trials of Chinese herbal medicines in China, noted that classical prescriptions, including Chuanxiong Cha Tiao San, Chuanxiong Ding Pain Drink, Dispersing Migraine Soup, Blood Mansions Expelling Blood Stasis Soup, and Tong Qiao Wu Blood Soup, as well as Chinese patent medicines, including Zheng Tian Wan, Headache Ning Capsules, Tong Tian Oral Liquid, and Nourishing Blood and Clearing Brain Granules (Pills), can all be used to treat migraine. In a multicenter randomized double-blind placebo-controlled trial in China, a greater percentage of headache attacks were reduced by > 50% after 12 weeks of treatment with Tianshu capsules than with a placebo [62.06% (422/680) versus 23.93% (56/234), *p* < 0.0001], and at the subsequent 4 weeks follow-up, the percentage of headache attacks decreased by > 50% in the Tianshu capsule group increased to 70.76% (392/554), with the percentage of headache attacks reduced by > 50% in the Tianshu capsule group (392/554), which was still greater than that in the placebo group [26.32% (60/228), *P* < 0.0001], suggesting that Tianshu is an effective, well-tolerated prophylactic drug that continues to have a preventive effect after discontinuation (Yu et al., 2019). A meta-analysis revealed that headache ninhydrin can be an effective treatment for migraine that is better than Western medicine alone and may yield better results when combined with Western medicine (Tan et al., 2014). In a study by Chai et al. (2022), the pharmacological mechanisms of Chuanxiong in the treatment of migraine included the following: the inhibition of oxygen free radical activity and the reduction of inflammation; the prevention of neuronal apoptosis and protection of brain nerves; the regulation of neurotransmitters and lowering of the pain threshold; and the prevention of platelet aggregation and the regulation of the intestinal flora. All the above studies have shown that some Chinese medicines have good efficacy in treating migraine. The most important issue to be studied in China is how to find and extract effective and stable components of relevant Chinese medicines and how to increase the efficacy of Chinese medicines internationally.

## 3 Summary and outlook

To understand the current profile of migraine patients in China, this paper reviews the incidence and disability of migraine in China over the past 10 years, as well as the new treatment advances that are of greatest concern to patients, on the basis of the migraine-related data in GBD2019.

The incidence of migraine in China slightly decreased from 2010 to 2017, followed by an increasing trend after 2017—a pattern consistent with global trends, although the increase was more pronounced in China. Combining the incidence rates of multiple SDI countries/regions, we again found that China’s incidence data were unusually low overall, and we therefore suspect that the increasing trend in migraine incidence in China after 2017 may be a manifestation of previously invisible data. The incidence of migraine is influenced mainly by sex and age. The sex ratio (male/female) of migraine incidence in China was significantly higher than that reported globally. The incidence of migraine in China is highest in the 10–14 years age group. The incidence rate is higher in the 5–49 years age group than overall, but the incidence rate in China is always lower than the global incidence rate in this age group, probably because a large proportion of patients in this age group are not seen and treated. These findings highlight the need to enhance care and support for migraine among women in China and to improve public awareness and understanding of this condition.

The trend in the ASDR for migraine in China aligns with the global pattern, demonstrating overall stability without significant changes from 2010 to 2019. However, the rate of increase in migraine-related disability in China, although not statistically significant, appears slightly more pronounced than the global average. This subtle difference may warrant further investigation and longer-term monitoring to determine whether it reflects an emerging trend. The severity of migraine, the presence of comorbid psychiatric disorders, the type of migraine, prodromal symptoms and the availability of ongoing treatment all influence the degree of disability in migraine patients.

In addition, social correlates of treatment for migraine patients in China need to be added to the study. New advances in the treatment of migraine in China can be classified into three main types: drug therapy, neuromodulation therapy and traditional Chinese medicine therapy. CGPR-related drugs are a hotspot of recent research on migraine drug therapy, but research on CGPR-related drugs in China is still in the initial stage. Recently, neuromodulation therapy has also been a hotspot of research at home and abroad, and this treatment has good prospects and deserves more research. Traditional Chinese medicine (TCM) is a complete treatment method with improved clinical effects, but there is still a long way to go before TCM is widely recognized in the international arena.

## References

[B1] An Z. Xu L. Shi B. (2022). Neuroregulatory mechanism of migraine and related treatment progress. *Practical J. Cardiac Cereb. Pneumal Vascular Dis.* 30 120–125. Available online at: https://link.cnki.net/urlid/13.1258.R.20220905.1759.002

[B2] Ashina M. Katsarava Z. Do T. Buse D. Pozo-Rosich P. Özge A. (2021). Migraine: Epidemiology and systems of care. *Lancet* 397 1485–1495. 10.1016/S0140-6736(20)32160-7 33773613

[B3] Blumenfeld A. Varon S. Wilcox T. (2011). Disability, HRQoL and resource use among chronic and episodic migraineurs: Results from the International Burden of Migraine Study(IBMS). *Cephalagia* 31 301–315. 10.1177/0333102410381145 20813784

[B4] Carod-Artal F. Irimia P. Ezpeleta D. (2012). Chronic migraine:definition, epidemiology, risk factors and treatment. *Rev. Neurol.* 54 629–637. Available online at: https://pubmed.ncbi.nlm.nih.gov/22573510/22573510

[B5] Celentano D. Linet M. Stewart W. (1990). Gender differences in the experience of headache. *Soc. Sci. Med.* 30 1289–1295. 10.1016/0277-9536(90)90309-G 2367875

[B6] Cephalalgia. (2018). Headache classification committee of the international headache society (IHS) The international classification of headache disorders, 3rd edition. *Cephalalgia* 38 1–211. 10.1177/0333102417738202 29368949

[B7] Chai J. Zhang Y. Lv Y. (2022). Research progress on the pharmacological mechanism of Chuanxiong rhizome in the treatment of migraine. *J. Liaoning Univer. Chinese Med.* 13:11. 10.13194/j.issn.1673-842x.2023.05.021

[B8] Cleveland W. S. Devlin S. J. (1988). Locally weighted regression: An approach to regression analysis by local fitting. *J. Am. Stat. Assoc.* 83 596–610. 10.1080/01621459.1988.10478639

[B9] David P. Kling J. Starling A. (2014). Migraine in pregnancy and lactation. *Curr. Neurol. Neurosci. Rep.* 14:439. 10.1007/s11910-014-0439-7 24604057

[B10] Fan G. Rui H. Li G. (2019). Clinical study of migraine with anxiety, depression and sleep disorders. *J. PLA Med.* 31 79–81.

[B11] Ferrari M. Goadsby P. Burstein R. Kurth T. Ayata C. Charles A. (2022). Migraine. *Nat. Rev. Dis. Primers* 8:2. 10.1038/s41572-021-00328-4 35027572

[B12] GBD 2019 Diseases and Injuries Collaborators. (2020). Global burden of 369 diseases and injuries in 204 countries and territories,1990-2019: A systematic analysis for the Global Burden of Disease Study 2019. *Lancet* 396 1204–1222. 10.1016/S0140-6736(20)30925-9 33069326 PMC7567026

[B13] Guo C. (2022). Pharmacological treatment of migraine and its mechanism of action. *Med. Information* 3 81–83. Available online at: https://cstj.cqvip.com/Qikan/Article/Detail?id=7107109440

[B14] Hou J. Pu S. Xu X. Lu Z. Wu J. (2022). Real-time ultrasound-guided stellate ganglion block for migraine:an observational study. *BMC Anesthesiol.* 22:78. 10.1186/s12871-022-01622-8 35331152 PMC8944155

[B15] Huang J. Wang X. Jin Y. Lou G. Yu Z. (2024). Trends and prescribing patterns of antimigraine medicines in nine major cities in China from 2018 to 2022: A retrospective prescription analysis. *J Headache Pain* 25:62. 10.1186/s10194-024-01775-6 38654177 PMC11036710

[B16] Jin Z. Shi L. Wang Y. Yang L. Shi Y. Shen L. (2013). Prevalence of headache among children and adolescents in Shanghai, China. *J. Clin. Neurosci.* 20 117–121. 10.1016/j.jocn.2012.02.020 23098390

[B17] Kuruvilla D. Mann J. Tepper S. Starling A. Panza G. Johnson M. (2022). Phase 3 randomized, double-blind, sham-controlled Trial of e-TNS for the Acute treatment of Migraine (TEAM). *Sci. Rep.* 12:5110. 10.1038/s41598-022-09071-6 35332216 PMC8948251

[B18] Leonardi M. Raggi A. (2019). A narrative review on the burden of migraine: When the burden is the impact on people’s life. *J. Headache Pain* 20:41. 10.1186/s10194-019-0993-0 31023226 PMC6734273

[B19] Liu B. Tan G. (2014). A longitudinal study of migraine patients’ condition, disability and quality of life. *Chongqing Med.* 43 2983–2985. Available online at: https://cstj.cqvip.com/Qikan/Article/Detail?id=662110346

[B20] Liu Q. He H. Yang J. Feng X. Zhao F. Lyu J. (2020). Changes in the global burden of depression from (1990). to 2017: Findings from the Global Burden of Disease study. *Psychiatr. Res.* 126 134–140. 10.1016/j.jpsychires.2019.08.002 31439359

[B21] Liu R. Yu S. He M. Zhao G. Yang X. Qiao X. (2013). Health-care utilization for primary headache disorders in China: A population-based door-to-door survey. *J. Headache Pain* 14:47. 10.1186/1129-2377-14-47 23731663 PMC3673891

[B22] Luo G. Ma Y. Gou J. (2012). Clinical study of migraine patients with anxiety/depression and functional disability. *Chinese J. Neuropsychiatric Disord.* 28 477–479. 10.3969/j.issn.1002-0152.2012.08.007

[B23] Ma P. Dong X. Qu Y. He Z. Yin T. Cheng S. (2021). A narrative review of neuroimaging studies in acupuncture for migraine. *Pain Res. Manag.* 2021:9460695. 10.1155/2021/9460695 34804268 PMC8598357

[B24] MacGregor E. Hackshaw A. (2004). Prevalence of migraine on each day of the natural menstrual cycle. *Neurology* 63 351–353. 10.1212/01.wnl.0000133134.68143.2e 15277635

[B25] Murray C. Lopez A. (2013). Measuring the global burden of disease. *N. Engl. J. Med.* 369 448–457. 10.1056/NEJMra1201534 23902484

[B26] National Institute for Health and Care Excellence. (2021). *Headaches in over 12s:diagnosis and management.* London: National Institute for Health and Care Excellence (NICE).

[B27] Pellesi L. Do T. Hougaard A. (2024). Pharmacological management of migraine: Current strategies and future directions. *Expert Opin. Pharmacother.* 25 673–683. 10.1080/14656566.2024.2349791 38720629

[B28] Pellesi L. Jedie B. Barhum F. Al-Abdullah S. Martelletti P. Xiao Z. (2025). Head-to-head relief: Ubrogepant, rimegepant, and zavegepant in migraine treatment. *Pain Manag.* 15 279–284. 10.1080/17581869.2025.2494494 40238598 PMC12118443

[B29] Pogoda J. Gross N. Arakaki X. Fonteh A. Cowan R. Harrington M. (2016). Severe headache or migraine history is inversely correlated with dietary sodium intake: NHANES 1999–2004. *Headache* 56 688–698. 10.1111/head.12792 27016121 PMC4836999

[B30] Seng E. Buse D. Klepper J. Mayson S. Grinberg A. Grosberg B. (2017). Psychological factors associated with chronic migraine and severe migraine-related disability:an observational study in a tertiary headache center. *Headache* 57 593–604. 10.1111/head.13021 28139000 PMC5378650

[B31] Smitherman T. Ward T. (2011). Psychosocial factors of relevance to sex and gender studies in headache. *Headache* 51 923–931. 10.1111/j.1526-4610.2011.01919.x 21631477

[B32] Somaa F. Degraaf T. Sack A. (2022). Transcranial magnetic stimulation in the treatment of neurological diseases. *Front. Neurol.* 13:793253. 10.3389/fneur.2022.793253 35669870 PMC9163300

[B33] Stewart W. Lipton R. Dowson A. (2001). Development and testing of the Migraine disability assessment (MI-DAS) questionnaire to assess headache related disability. *Neurology* 56 S20–S28. 10.1212/wnl.56.suppl_1.s20 11294956

[B34] Stewart W. Lipton R. Celentano D. Read M. (1992). Prevalence of migraine headache in the United States: Relation to age, income, race, another sociodemographic factors. *JAMA* 267 64–69. 1727198

[B35] Takeshima T. Wan Q. Zhang Y. Komori M. Stretton S. Rajan N. (2019). Prevalence, burden, and clinical management of migraine in China, Japan, and South Korea: A comprehensive review of the literature. *J. Headache Pain* 20:111. 10.1186/s10194-019-1062-4 31805851 PMC6896325

[B36] Tan H. Mao S. Hu R. (2014). Meta-analysis of the efficacy and safety of cephalalalgene in the treatment of migraine. *China Med. Herald* 30 65–69.

[B37] Thuraiaiyah J. Erritzoe-jervld M. Al-khazalih M. Al-Khazali H. Schytz H. Younis S. (2022). The role of cytokines in migraine: A systematic review. *Cephalalgia* 42 1565–1588. 10.1177/03331024221118924 35962530

[B38] U.S. Food and Drug Administration (2013). *U.S. Food and Drug Administration De Novo Summary (K (1305). 56).* Available online at: https://www.accessdata.fda.gov/cdrh_docs/reviews/k130556.pdf (accessed 5 March, 2013)

[B39] Wang Y. Huang X. Yue S. (2022). Secular trends in the incidence of migraine in China from (1990). to 2019: A joinpoint and age-period-cohort analysis. *J. Pain Res.* 15 137–146. 10.2147/JPR.S337216 35058715 PMC8765540

[B40] Xia G. Zhang Z. (2014). Sleep quality survey and correlation analysis of anxiety and depression in 167 migraine patients. *China J. Med.* 16 616–617. Available online at: https://qikan.cqvip.com/Qikan/Article/Detail?id=50177910

[B41] Xingkai A. (2021). *Analysis of migraine susceptibility genes in Chinese Han Chinese.* China: Xiamen University. 10.27424/d.cnki.gxmdu.2019.000667

[B42] Xu J. Peng H. He B. (2021). Relationship between different prodromal symptoms and prognosis of migraine and its early warning value for headache. *Chin. Clin. Med.* 28 192–197. Available online at: https://qikan.cqvip.com/Qikan/Article/Detail?id=7104734229

[B43] Xu Z. Jia M. Liang X. Jing-Jing W. Guo-Jing F. Lin L. (2020a). Clinical practice guidelines for Chinese medicine in migraine (Draft for comment). *Chinese J. Traditional Chinese Med.* 45 5057–5067. 10.19540/j.cnki.cjcmm.20200903.502 33350221

[B44] Xu S. Yu L. Luo X. Wang M. Chen G. Zhang Q. (2020b). Manual acupuncture versus sham acupuncture and usual care for prophylaxis of episodic migraine without aura: Multicentre, randomised clinical trial. *BMJ* 368:m697. 10.1136/bmj.m697 32213509 PMC7249245

[B45] Yang J. (2022). Pharmacological effects and clinical evaluation of atogepant, a new drug for migraine. *Chin. J. Clin. Pharmacol.* 28 1950–1953. 10.13699/j.cnki.1001-6821.2022.16.024

[B46] Yang Y. Zhao H. Lu H. (2018). Analysis of headache impact and disability in patients with chronic migraine. *J. Pract. Med.* 34 4093–4098. 10.3969/j.issn.1006-5725.2018.24.019

[B47] Yin Y. Xu Y. (2010). Advances in genetics of migraine. *Chin. J. Neuropsychiatr. Disord.* 36 697–698. Available online at: https://cstj.cqvip.com/Qikan/Article/Detail?id=36194712

[B48] Yu J. Zhang R. (2022). Research progress on the mechanism of action of cerebellopontine nucleus electrical stimulation in the treatment of related diseases. *Shandong Med.* 62 101–104. Available online at: https://qikan.cqvip.com/Qikan/Article/Detail?id=7108124601

[B49] Yu S. Liu R. Zhao G. Yang X. Qiao X. Feng J. (2012). The prevalence and burden of primary headaches in China: A population-based door-to-door survey. *Headache* 52 582–591. 10.1111/j.1526-4610.2011.02061.x 22590713

[B50] Yu S. Ran Y. Xiao W. Tang W. Zhao J. Chen W. (2019). Treatment of migraines with Tianshu capsule: A multi center, double blind, randomized, placebo controlled clinical trial. *BMC Complement. Altern. Med.* 19:370. 10.1186/s12906-019-2775-2 31842860 PMC6915862

[B51] Yu S. Zhang Y. Yao Y. Cao H. (2020). Migraine treatment and healthcare costs: Retrospective analysis of the China health insurance research association (CHIRA) database. *J. Headache Pain* 21:53. 10.1186/s10194-020-01117-2 32404048 PMC7222520

[B52] Yuan H. Chuang T. (2021). Update of neuromodulation in chronic migraine. *Curr. Pain Headache Rep.* 25:71. 10.1007/s11916-021-00988-7 34766212 PMC8583582

[B53] Zhang B. Li B. Qiu Z. Dong X. (2017). Advances in migraine research. *Asia-Pacific Traditional Med.* 13 43–45. Available online at: https://cstj.cqvip.com/Qikan/Article/Detail?id=673857586

[B54] Zhang X. Huang X. J. (2016). A clinical study of migraine with anxiety/depression and functional disability. *Chinese J. Pract. Neurol. Disord.* 19 69–71. Available online at: https://cstj.cqvip.com/Qikan/Article/Detail?id=671618114

[B55] Zhao H. Xiao Z. Zhang L. Ford J. Zhong S. Ye W. (2023). Real-world treatment patterns and outcomes among patients with episodic migraine in China: Results from the Adelphi migraine disease specific programme™. *J. Pain Res.* 6 357–371. 10.2147/JPR.S371887 36762367 PMC9904300

[B56] Zhao L. Chen J. Li Y. Sun X. Chang X. Zheng H. (2017). The long term effect of acupuncture for migraine prophylaxis: A randomized clinical trial. *JAMA Intern Med.* 177:508515. 10.1001/jamainternmed.2016.9378 28241154

[B57] Zhou Z. Yu S. (2015). Migraine related assessment tools. *Chin. J. Pain Med.* 21 241–244. Available online at: https://cstj.cqvip.com/Qikan/Article/Detail?id=665112427

