# Supplementary material for: A summary of the current circumstances of migraineurs in China: a review of the GBD2019 database
Source: Front Hum Neurosci. 2025 Oct 10;19:1626607. doi: 10.3389/fnhum.2025.1626607 (PMC12549686; doi:10.3389/fnhum.2025.1626607)
Supplement: Supplementary file 1 [file Table_1.doc]

The search keywords was tailored to each section:

Part 1 (Incidence): ("epidemiology"[MeSH Subheading] OR "epidemiology"[All Fields] OR "incidence"[All Fields] OR "incidence"[MeSH Terms] OR "incidences"[All Fields] OR "incident"[All Fields] OR "incidents"[All Fields]) AND ("migrain"[All Fields] OR "migraine disorders"[MeSH Terms] OR ("migraine"[All Fields] AND "disorders"[All Fields]) OR "migraine disorders"[All Fields] OR "migraine"[All Fields] OR "migraines"[All Fields] OR "migraine s"[All Fields] OR "migraineous"[All Fields] OR "migrainers"[All Fields] OR "migrainous"[All Fields])

Part 2 (Disability):("disabilities"[All Fields] OR "disability"[All Fields] OR "disablement"[All Fields] OR "disablements"[All Fields] OR "disabling"[All Fields] OR "disablity"[All Fields] OR "persons with disabilities"[MeSH Terms] OR ("persons"[All Fields] AND "disabilities"[All Fields]) OR "persons with disabilities"[All Fields] OR "disabled"[All Fields]) AND ("migrain"[All Fields] OR "migraine disorders"[MeSH Terms] OR ("migraine"[All Fields] AND "disorders"[All Fields]) OR "migraine disorders"[All Fields] OR "migraine"[All Fields] OR "migraines"[All Fields] OR "migraine s"[All Fields] OR "migraineous"[All Fields] OR "migrainers"[All Fields] OR "migrainous"[All Fields]).

Part 3 (Teatment):("therapeutics"[MeSH Terms] OR "therapeutics"[All Fields] OR "treatments"[All Fields] OR "therapy"[MeSH Subheading] OR "therapy"[All Fields] OR "treatment"[All Fields] OR "treatment s"[All Fields]) AND ("migrain"[All Fields] OR "migraine disorders"[MeSH Terms] OR ("migraine"[All Fields] AND "disorders"[All Fields]) OR "migraine disorders"[All Fields] OR "migraine"[All Fields] OR "migraines"[All Fields] OR "migraine s"[All Fields] OR "migraineous"[All Fields] OR "migrainers"[All Fields] OR "migrainous"[All Fields]) AND ("china"[MeSH Terms] OR "china"[All Fields] OR "chinese"[All Fields])
