# Supplementary material for: A summary of the current circumstances of migraineurs in China: a review of the GBD2019 database
Source: Front Hum Neurosci. 2025 Oct 10;19:1626607. doi: 10.3389/fnhum.2025.1626607 (PMC12549686; doi:10.3389/fnhum.2025.1626607)
Supplement: Supplementary file 2 [file Table_2.doc]

Histogram and Q‒Q plot of residuals for China. The residuals are symmetrically distributed around zero and show no substantial deviation from normality, suggesting that the LOWESS model adequately captured the temporal trend of China’s ASIR data.


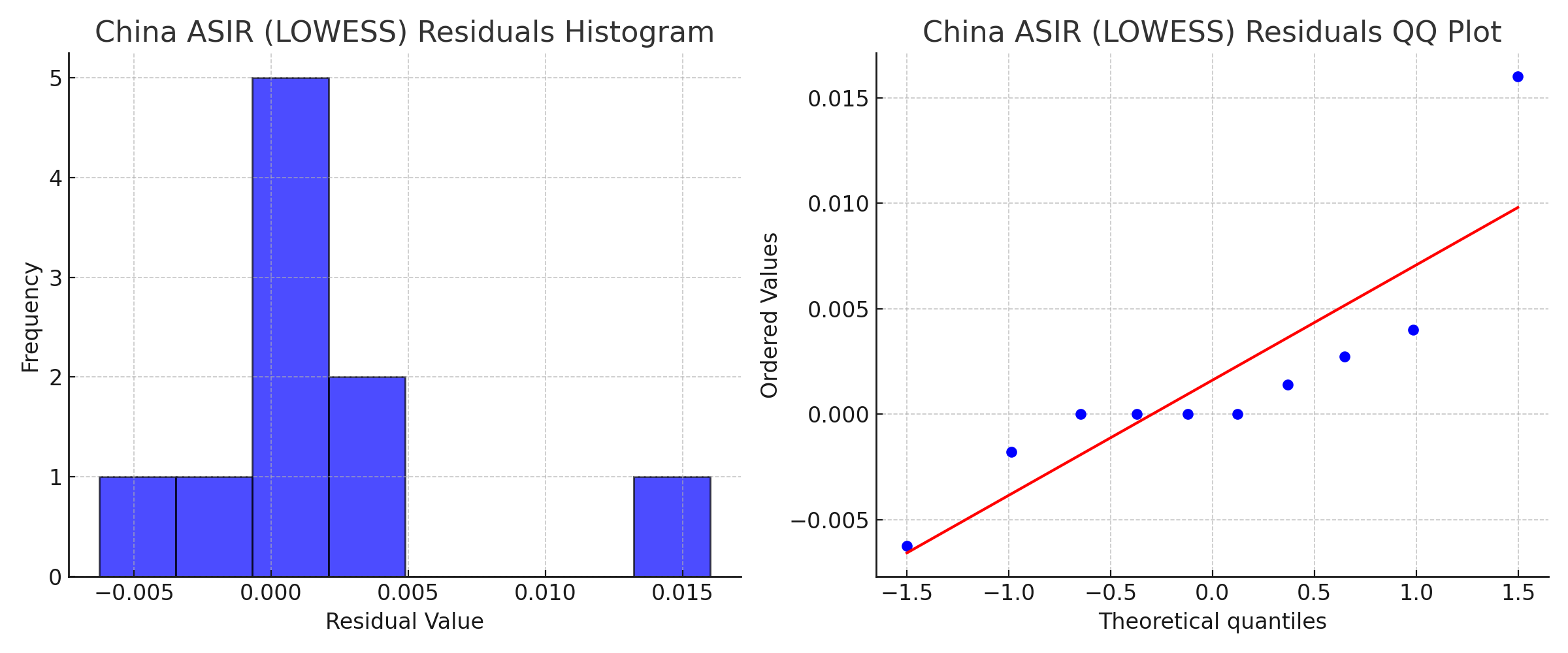


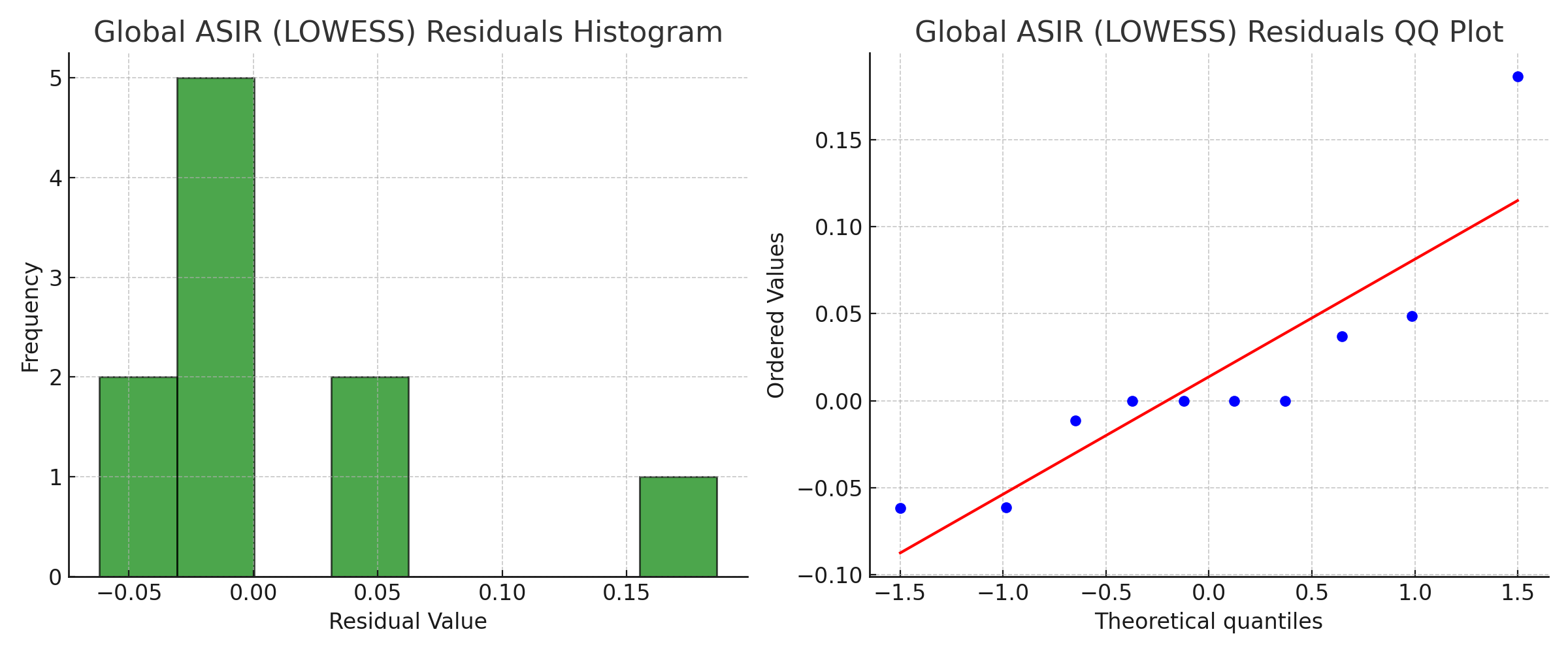
Histogram and Q‒Q plot of residuals for Global ASIR. The residuals exhibit slightly higher variability compared with China but remain approximately normally distributed without systematic bias, supporting the appropriateness of the LOWESS model for global data.
